# Higher locomotor costs of pregnancy in viviparous compared to oviparous common lizards (*Zootoca vivipara*)

**DOI:** 10.1002/ece3.70171

**Published:** 2024-08-16

**Authors:** Robert Hussain, Hans Recknagel, Kathryn R. Elmer

**Affiliations:** ^1^ School of Biodiversity, One Health & Veterinary Medicine, College of Medical, Veterinary & Life Sciences University of Glasgow Glasgow UK; ^2^ Present address: Biotechnical Faculty, Department of Biology University of Ljubljana Ljubljana Slovenia

**Keywords:** costs of reproduction, life history, oviparity, squamate, trade‐offs, viviparity

## Abstract

Pregnancy is a physiological cost of reproduction for animals that rely on fleeing to avoid predators. Costs of reproduction are predicted to differ between alternative reproductive strategies or modes, such as egg‐laying (oviparity) or live‐bearing (viviparity). However, disentangling the factors that comprise this cost and how it differs for oviparous or viviparous females is challenging due to myriad environmental, biological, and evolutionary confounds. Here, we tested the hypothesis that costs of pregnancy differ between oviparous and viviparous common lizards (*Zootoca vivipara*). We predicted that the degree of locomotor impairment during pregnancy and therefore the cost of reproduction would be higher in viviparous females. We conducted our experiment in a hybrid zone containing oviparous and viviparous common lizards. Due to the common environment and inclusion of hybrid individuals, we could infer whether differences were inherent to parity mode. We found that the average and maximum running speed of pregnant females was slower than after they had given birth or laid eggs. Viviparous females experienced an additional pregnancy weight burden and for a longer time period, but were not slower at running than pregnant oviparous females. In addition, we found a parity mode‐specific effect of reproductive investment; producing larger clutches was costlier for the locomotor performance of viviparous females for reasons other than the mass increase. Locomotor costs were found to be intermediate in hybrid females, indicating that they are specific to each reproductive mode. Our study shows that viviparous females experience an additional physical and physiological cost of pregnancy and reproductive investment. This two‐fold cost implies that viviparous females modulate resource allocation decisions and/or adjust their behavioural responses that result from locomotor impairment.

## Graphical abstract

In a powerful design controlling phylogeny and environment, we show that pregnant females experience higher locomotor costs for viviparity than oviparity, for reasons other than the mass increase of clutch size. Performance costs are consistently intermediate in hybrid females, demonstrating they are inherent to the reproductive mode.

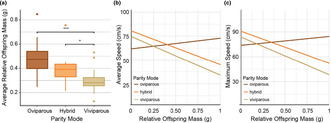

## 1 INTRODUCTION

Life history theory predicts that variation in phenotypic traits exists because limited energetic resources compel organisms to balance trade‐offs in investment between these traits (Husak & Lailvaux, 2022; Lailvaux & Husak, 2017). Classical life history phenotypes refer to traits such as age of first reproduction, fecundity, longevity, gestational period, somatic maintenance, and reproductive investment (Husak et al., 2016; Lailvaux & Husak, 2014). In the case of reproductive investment, trait variation is predicted to arise as a consequence of the trade‐off between an organism's current investment compared to potential future reproductive events (Reznick, 1985). This implies that there are ‘costs of reproduction’, whereby a greater initial investment may reduce the probability of surviving to reproduce again or may reduce future fecundity (Qualls & Shine, 1998). A survival cost is when current reproduction reduces an individual's chance of living to reproduce again, for example, due to behavioural and physiological changes during the reproductive period that diminish body condition and increase predation risk (Miles et al., 2000). A fecundity cost is experienced when reproduction reduces the energy available for future reproductive episodes (Shine, 1980). Because the energy obtained from food is directed to many different functions such as storage, growth, and reproduction, organisms must prioritize the allocation of energy to one function at the expense of another (Sorci et al., 1995).

During reproductive periods, organisms display behaviours that result in either or both of these types of reproductive costs, and even trade‐offs between them (Qualls & Shine, 1998). For example, a pregnant lizard that increases time spent basking may incur both a survival cost due to increased predation risk as well as an energy cost due to increased metabolic energy expenditure (Qualls & Shine, 1998; Shine, 1980). Additionally, resource allocation decisions may be influenced by environmental conditions and by the inherent characteristics of particular reproductive strategies. For example, in environments with plentiful food conditions, it is thought that viviparity would be favoured over oviparity (Buddle et al., 2019; Trexler & DeAngelis, 2003).

Costs can be difficult to quantify directly, so a useful indirect measure of reproductive cost is the locomotor cost (Le Galliard et al., 2003; Olsson et al., 2000; Shine, 2003b). Locomotor ability is essential for the survival of many animals, both vertebrate and invertebrate (Ru et al., 2017): it is important for activities such as locating food, avoiding predation, and finding mates, which all affect the overall lifetime reproductive success of individual females (Le Galliard et al., 2003). Consequently, a decrease in locomotor ability likely has an associated survival (e.g., reduced capacity to escape predators) or energy (e.g., reduction in foraging success) cost (Le Galliard et al., 2003).

Performance traits can be defined as a measure of how well an organism can perform an ecologically relevant task (Irschick et al., 2008; Irschick & Garland, 2001; Lailvaux & Husak, 2014; Lailvaux & Irschick, 2006). Performance traits are affected by many morphological, physiological, and biochemical factors such as temperature, habitat composition, presence/absence of food in the stomach, limb length, parasite/disease burden, hydration status, and reproductive state (Garland & Losos, 1994). The link from performance to fitness was first proposed by Arnold's (1983) ecomorphological paradigm, which assumes that variation in an organism's morphological trait is related to variation in its maximal performance ability of an important ecological trait. This can then be linked to a measure of fitness such as reproductive success (Garland & Losos, 1994; Husak & Fox, 2008; Irschick & Garland, 2001) or survival (Irschick et al., 2008). As performance traits are energetically costly to maintain and express, there is likely to be trade‐offs between these traits and other life‐history traits (i.e., performance‐life history trade‐offs—Husak et al., 2016; Husak & Lailvaux, 2022; Lailvaux & Husak, 2014). As a consequence of energetic allocation to these traits at the expense of reproductive traits, this could be considered another ‘cost of reproduction’. For example, increasing investment in locomotor performance by training green anole lizards (*Anolis carolinensis*) reduced their reproductive output (Husak et al., 2016).

Locomotor ability is a performance trait that is relatively easily measured and can be defined in terms of maximal sprint speed and/or endurance as well as acceleration (burst speed), deceleration, and manoeuvrability (Higham, 2007), for example (Garland & Losos, 1994; Husak et al., 2016; Husak & Lailvaux, 2022; Lailvaux & Husak, 2017). Locomotor costs due to pregnancy are widely experienced by animals with internal gestation, both invertebrates (Shaffer & Formanowicz, 1996) and vertebrates (Orr & Garland, 2017; see references therein). In gravid females, decreased locomotor ability is usually due to the weight burden of the offspring she is carrying: the mass and volume of the developing embryos or eggs (Cox & Calsbeek, 2009; Shine, 2003b). This elevated body mass increases the energy required for movement and reduces manoeuvrability (Le Galliard et al., 2003), unless females possess compensatory modifications that offset the added weight stress (Scales & Butler, 2007). Additionally, it is thought that physiological effects of pregnancy may alter locomotor ability in other ways, irrespective of weight, for example, due to reduced metabolism or muscle loss (Olsson et al., 2000). For these reasons, squamate reptiles in particular have emerged as a useful model system to investigate locomotor costs due to pregnancy (Cooper et al., 1990; Dayananda et al., 2017; Goodman, 2006; Miles et al., 2000; Olsson et al., 2000; Scales & Butler, 2007; Seigel et al., 1987; Shine, 1980, 2003a; Sinervo et al., 1991; Van Damme et al., 1989; Webb, 2004; Winne & Hopkins, 2006). Squamate species can be oviparous (egg‐laying) or viviparous (live‐bearing) and are therefore valuable for comparing the costs of pregnancy between reproductive (parity) modes (Blackburn, 2006; Bleu et al., 2012). Given equal reproductive output in offspring number, theory predicts that viviparous females suffer a greater locomotor cost compared to oviparous females due to their (i) increased length of pregnancy and (ii) increased offspring weight as a result of transfer of water, gas, and nutrients during gestation (Qualls & Andrews, 1999; Qualls & Shine, 1998; Recknagel & Elmer, 2019).

The common lizard (*Zootoca vivipara*) is a distinctly useful model organism for investigating the costs of pregnancy and how they differ between parity modes because it is one of the few amniotes that has conspecific oviparous and viviparous lineages, that is, reproductively bimodal (Bleu et al., 2012; Recknagel et al., 2021; Recknagel & Elmer, 2019). Reproductive traits within parity modes are likely influenced by environmental variation across the range, while parity mode is fixed within lineages, which are usually allopatric (Bleu et al., 2012; Heulin et al., 1991, 2000). Unusually, in the Carinthian Alps, oviparous and viviparous common lizards come into contact and occasionally interbreed, producing hybrid females that display intermediate parity mode traits (Lindtke et al., 2010; Recknagel et al., 2021; Recknagel & Elmer, 2019). These hybrid females oviposit thin shelled eggs containing embryos at a developmental stage intermediate between pure oviparous and pure viviparous females (McLennan et al., 2019; Recknagel et al., 2021). At this altitude, viviparous and oviparous females usually have a single litter or clutch per year (Recknagel & Elmer, 2019; Rodríguez‐Díaz & Braña, 2012) and viviparous females give birth to one fewer offspring per clutch on average compared to oviparous females (six vs. seven), though clutch size can vary considerably (Recknagel & Elmer, 2019). These populations can therefore be studied for costs of pregnancy between the different parity modes whilst minimizing confounding factors related to environment and phylogeny.

The current study aims to quantify the functional cost of pregnancy in oviparous and viviparous female lizards, evaluated by the traits of locomotor performance and reproductive investment. Importantly, we assess inherent characteristics of parity mode while controlling for environment because we include oviparous, viviparous, and hybrid females that co‐occur at a single site (Lindtke et al., 2010; Recknagel & Elmer, 2019; Recknagel et al., 2023). We tested the hypothesis that viviparous lizards experience a higher reproductive burden than oviparous lizards and that this negatively affects their locomotor ability. To do so, we measured the effect of pregnancy on locomotor traits that are likely to affect lizard survival: sprint speed and distance travelled. Additionally, we measured reproductive investment for each female and assessed how this affected running performance during and after pregnancy. Our predictions were: (i) there is a fundamental cost to locomotion experienced by pregnant females due to increased mass; (ii) viviparous lizards suffer an increased cost of locomotion during pregnancy; and (iii) hybrid females exhibit intermediate locomotor and reproductive costs, if these costs are genetically determined by reproductive mode.

## 2 MATERIALS AND METHODS

### 2.1 Sampling

Pregnant female common lizards were captured from the Gailtal region, Austria, between May and August 2016: 45 oviparous, 47 viviparous, and 14 hybrid individuals. Sex was identified by the absence of a penile bulge in females, and pregnancy was inferred by the presence of mating bite marks at collection, followed by monitoring all females in captivity to oviposition/parturition in the experiment. Parity mode was established by phenotype (offspring developmental stage at oviposition/parturition and eggshell thickness) and genotype (SNPs generated from ddRADSeq data; following McLennan et al. (2019)) for these individuals. Husbandry conditions followed Recknagel and Elmer (2019).

Snout to vent length (SVL; in mm) and tail length (mm) of each female was recorded upon capture. Data on the number of offspring (clutch size) and relative offspring mass (ROM) was recorded at parition (= oviposition or parturition; Blackburn, 2000). ROM is the sum of the mass of the successfully hatched/live‐born offspring (excluding eggshell, amniotic fluid, and yolk) divided by the mass of the female after parition (see Recknagel & Elmer, 2019 for details).

### 2.2 Running speed trial design

A female was placed in a rectangular tank (0.8 m length) near the sampling site, with a natural sediment base (collected from sample sites) and a measuring tape on the longer side. An aerial‐view digital camera was set up to record running. Lizards were approached posteriorly by hand to trigger a flight response. The tank size was based on experience with the escape behaviour of this species, which is short and intermittent (Bauwens & Thoen, 1981). As temperature is known to affect locomotor performance, females were incubated in a box placed into an Exo Terra Thermoelectric Reptile Egg Incubator for 30 min at their optimum temperature (32°C) prior to a running trial (Van Damme et al., 1989, 1990).

A running trial consisted of the three fastest recorded runs within a day, and this was done over (i) four consecutive days whilst pregnant and (ii) four consecutive days after parition. The three fastest runs within a day were included to provide an average of the maximal sprinting speed of the lizards (as well as a record of their maximal performance), and these were measured over 4 days to account for potential variation in individual motivation (Garland & Losos, 1994; Losos et al., 2002). Individual weight was recorded before every trial using a Smart Weigh high precision scale (to the nearest 0.001 g). Pregnancy progress was recorded for each trial as the difference between the date of the trial and the date of parition. Running trials were conducted between June and August.

### 2.3 Video analysis

Videos were analysed on an iMac computer using Quicktime Player. To calculate running speed, the distance travelled was measured using a ruler on the screen marked with lines every 5 mm and standardized to the tank measuring tape in each video. The number of frames during a run was recorded to calculate the time taken to cover the running distance (60 frames = 1 s). The speed (cm/s) was then calculated by dividing the distance (in centimetres) by time (in seconds). In each trial, the three fastest runs were retained, and the average (mean) speed from each day was used to calculate the overall average velocity for each individual lizard during the 4 days of pregnancy and 4 days post‐parition. The average maximum speed of each female was recorded by taking the mean of the fastest run each day over the 4 days during pregnancy and post‐parition. The average distance run by each female during pregnancy and post‐parition was obtained by taking the average distance travelled in each trial and then calculating the average over the 4 days separately. The maximum distance run by each lizard per running trial was also recorded, and an average maximum distance was calculated for the trials while pregnant and trials post‐parition.

### 2.4 Statistical analysis

Data were analysed using R software version 4.3.1 (R Core Team, 2023). A linear mixed‐effects model (LMM) was first constructed to investigate the effect of parity mode (factor with three levels—oviparous, hybrid, and viviparous) and pregnancy status (factor with two levels—pregnant and post‐parition) on the average mass of the lizard (average mass model). This model included an interaction between parity mode and pregnancy status, snout‐vent length (SVL), and trial number (factor with four levels—day 1, day 2, day 3, and day 4). Because lizard mass during pregnancy may depend on the pregnancy progress (i.e., the time between ovulation and parition), we ran an additional model testing for an effect of parity mode, snout‐vent length (SVL), trial number, and pregnancy progress on the mass during pregnancy (a subset of the data). Next, linear models were constructed to assess if clutch size (number of offspring) and relative offspring mass (ROM) differed by parity mode for pregnant females. These linear models were performed on a subset of the data consisting of only the pregnant females with ROM data (34 oviparous, 42 viviparous, and eight hybrids) and clutch size data (42 oviparous, 46 viviparous, and 14 hybrids), respectively. The Shapiro‐Wilk test was used to assess the normality of the residuals of these models. Due to violations of normality, these models were altered (see Section 2.3 above). Multiple pairwise comparisons were performed to test for differences between the levels of the factor variables using the ‘emmeans’ package in R (Lenth, 2022).

LMMs were then constructed to test the effect of parity mode (factor with three levels—oviparous, hybrid, and viviparous), pregnancy status (factor with two levels—pregnant and post‐parition), relative offspring mass (ROM), clutch size, lizard mass, trial number (factor with four levels—day 1, day 2, day 3, and day 4), snout‐vent length (SVL), and tail length on the mean speed, maximum speed, mean distance travelled, and the maximum distance travelled. These models used data from 85 female lizards—36 oviparous, 41 viviparous, and eight hybrids. Other morphological traits that may be important for speed, such as limb length, do not vary considerably among female common lizards (Guillaume et al., 2006; Horváthová et al., 2013), and while alternative parity modes show clear differences in genetics, they are basically indistinguishable based on morphology (Guillaume et al., 2006; Recknagel et al., 2021). Here, the variables lizard mass, SVL, and tail length were included to account for the main confounding effects of body size on lizard running speed. In all LMMs, lizard ID was treated as a random effect to account for the non‐independent nature of the data. Model selection was conducted using likelihood ratio tests (LRTs). To directly test whether the effect of pregnancy differs by parity mode, the most complex model included a pregnancy status × parity mode interaction. In addition, models included a ROM × parity mode interaction to assess if the effect of relative offspring mass on the locomotor variables varied by parity mode. ANOVA tests were run on each final LMM using the ‘anova’ command in R; estimated *F*‐values, *p*‐values, and associated degrees of freedom (rounded to whole integers) were obtained using Satterthwaite's method (Type 3) from the ‘lmerTest’ package (Kuznetsova et al., 2017).

## 3 RESULTS

### 3.1 Effect of pregnancy on mass and speed

**FIGURE 1:**
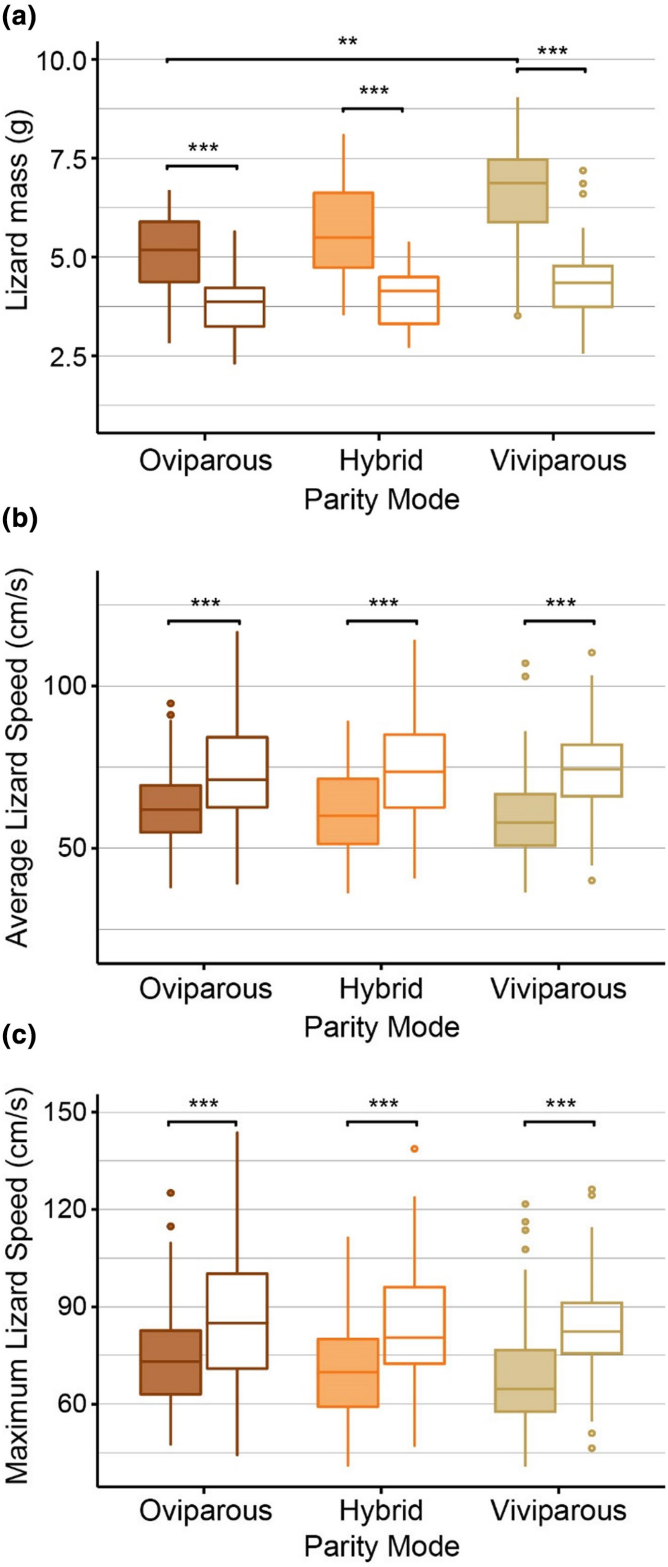
Average mass (a), average speed (b), and maximum speed (c) of female common lizards (*Zootoca vivipara*) during and after pregnancy (*N* = 106: 45 oviparous, 47 viviparous, and 14 hybrids). Boxplots show the median value (thick line) and upper and lower quartiles, as well as the range. Filled shapes refer to during pregnancy, and empty refers to post‐parition.

We found a significant interaction between parity mode and pregnancy status: the effect of pregnancy on female mass differed between parity modes as inferred from the average mass model (*F*_2,708_ = 85.65, *p* < .001). During pregnancy, viviparous females were found to be significantly heavier (6.54 ± 1.30 g) than oviparous females (5.09 ± 1.00 g), with hybrids showing intermediate values (5.66 ± 1.27 g) between oviparous and viviparous (Figure 1a). Snout‐vent length was also found to significantly affect lizard mass, with increasing SVL resulting in heavier lizards (*F*_1,100_ = 142.01, *p* < .001). Lizard mass was not affected by trial number (*F*_1,702_ = 0.12, *p* > .1), but pregnant females of all parity modes increased in mass from the time of capture until parition (*F*_1,99_ = 6.95, *p* < .01).

Pregnancy affected both the average and the maximum sprint speed (average speed *F*_1,655_ = 14.644, *p* < .001; maximum speed *F*_1,649_ = 12.30, *p* < .001). For all parity modes, females were slower when pregnant (average speed: oviparous 62.5 ± 11.2 cm/s, viviparous 59.5 ± 16.1 cm/s, hybrids 61.5 ± 13.2 cm/s) compared to post‐parition (average speed: oviparous 72.8 ± 16.1 cm/s, viviparous 74.6 ± 11.8 cm/s, hybrids 74.1 ± 15.0 cm/s; Figure 1b,c; Table 1). There was also found to be no significant interaction between pregnancy status and parity mode: the effect of pregnancy did not vary due to parity mode for average running speed (2ΔLL = 0.587, df = 2, *p* > .1) or maximum running speed (2ΔLL = 0.034, df = 2, *p* > .1). Additionally, there was no significant effect of pregnancy or parity mode on the average or maximum distance travelled (Figure S1).

**TABLE 1:** Summary of final linear mixed effect models.

Model	Variable	Fixed effects	Estimate	Standard error	*t*‐value	*p*‐value
Average speed		Intercept (oviparous post‐parition)	7.57	14.57	0.52	.604
	Pregnancy Status	Pregnant	−6.05	1.58	−3.83	**<.001**
	ROM	ROM	11.13	12.76	0.87	.386
	ROM × parity mode	Hybrid	−46.50	24.28	−1.92	.059
		Viviparous	−51.60	22.43	−2.3	**.024**
	Parity Mode	Hybrid	18.77	11.25	1.67	.099
		Viviparous	12.85	8.60	1.49	.139
	Mass	Mass	−4.15	0.72	−5.79	**<.001**
	Trial Number	Trial No. 2	0.25	1.05	0.24	.810
		Trial No. 3	0.43	1.05	0.41	.686
		Trial No. 4	−2.85	1.05	−2.70	**.007**
	Snout‐Vent Length	SVL	1.32	0.27	4.90	**<.001**
Maximum speed		Intercept (oviparous post‐parition)	20.01	17.15	1.17	.246
	Pregnancy Status	Pregnant	−7.19	2.05	−3.51	**<.001**
	ROM	ROM	10.64	14.87	0.72	.476
	ROM × parity mode	Hybrid	−49.12	28.28	−1.74	.086
		Viviparous	−55.98	26.18	−2.14	**.036**
	Parity Mode	Hybrid	16.95	13.11	1.29	.200
		Viviparous	9.91	10.03	0.99	.326
	Mass	Mass	−4.57	0.93	−4.94	**<.001**
	Trial Number	Trial No. 2	0.03	1.39	0.02	.981
		Trial No. 3	−0.05	1.39	−0.03	.974
		Trial No. 4	−3.50	1.39	−2.51	**.012**
	Snout‐Vent Length	SVL	1.37	0.32	4.28	**<.001**
Average distance		Intercept	14.15	77.05	0.184	.855
	Mass	Mass	−13.50	2.05	−6.58	**<.001**
	Trial Number	Trial No. 2	−3.63	6.00	−0.60	.546
		Trial No. 3	−10.33	6.02	−1.72	.087
		Trial No. 4	−27.01	6.02	−4.49	**<.001**
	Snout‐Vent Length	SVL	6.29	1.30	4.86	**<.001**
Maximum distance		Intercept (oviparous post‐parition)	145.31	88.23	1.65	.102
	Mass	Mass	−8.98	2.56	−3.51	**<.001**
	Trial Number	Trial No. 2	−10.01	7.53	−1.33	.184
		Trial No. 3	−12.06	7.55	−1.60	.111
		Trial No. 4	−36.92	7,55	−4.89	**<.001**
	Snout‐Vent Length	SVL	5.26	1.49	3.53	**<.001**

*Note*: Fixed effect estimates obtained from the LMMs generated (see Methods). Only the final, best model is shown for each of the four tested models. Data taken from 85 female lizards (36 oviparous, 41 viviparous, and eight hybrids). Estimates of each fixed effect alongside interaction terms and associated standard errors are shown (intercept is oviparous post‐parition). Fixed effects returning significant *p*‐values (*p* < .05) highlighted in bold.

### 3.2 Effect of reproductive investment

**FIGURE 2:**
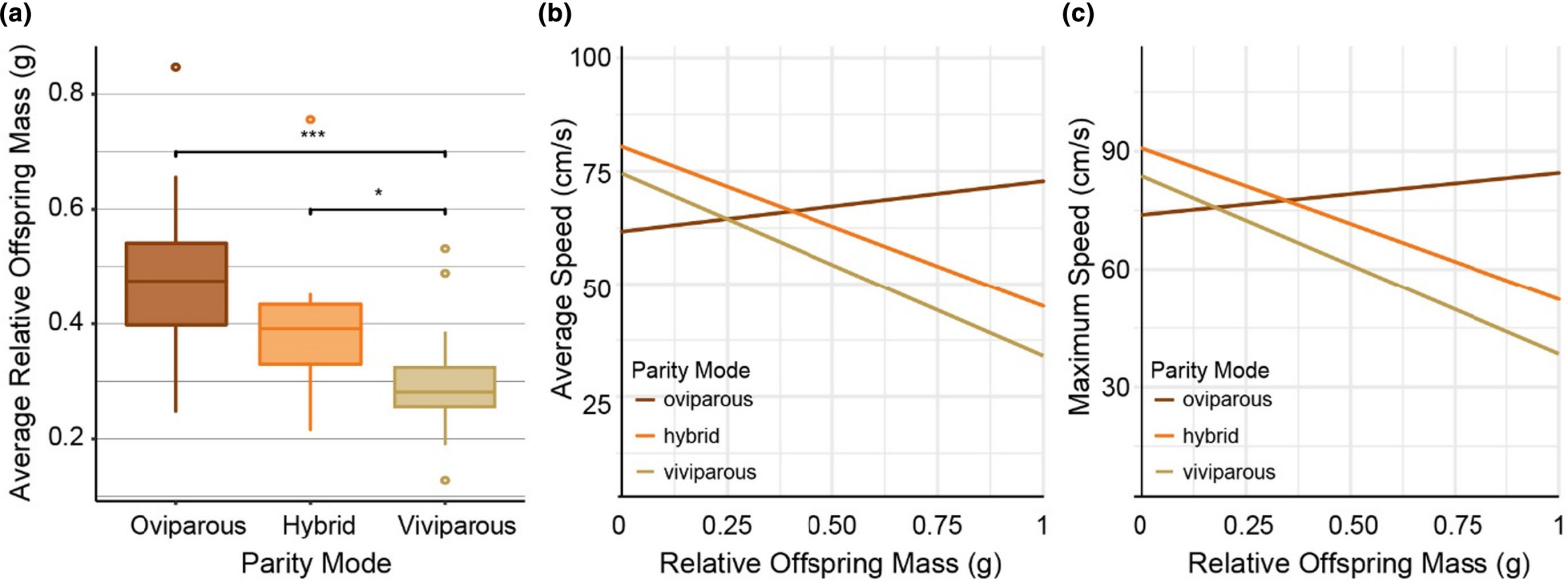
Effect of reproductive investment on parity mode and speed (*N* = 84; 34 oviparous, 42 viviparous, and eight hybrid lizards). (a) Average relative offspring mass (ROM) for each parity mode. (b) Predicted relationship between average speed (cm/s) and relative offspring mass (g) of female common lizards obtained from LMM. (c) Predicted relationship between maximum speed (cm/s) and relative offspring mass (g) of female common lizards obtained from LMM.

The models testing the effect of parity mode on relative offspring mass (total offspring mass relative to mother's mass) and clutch size were modified as both of these models resulted in residuals with non‐normal distributions (Shapiro‐Wilk *p*‐values = .00053 and .0254, respectively). ROM was log transformed and re‐modelled with the same variables as before, and clutch size was re‐modelled using a generalized linear model with a Poisson distribution. ROM was found to differ between parity modes (*F*_2,81_ = 34.68, *p* < .001) and was highest for oviparous (mean ± SD = 0.482 ± 0.116), intermediate for hybrids (mean ± SD = 0.407 ± 0.162) and lowest for viviparous (mean ± SD = 0.295 ± 0.074; Figure 2a). Pairwise comparisons revealed that the ROM of viviparous females was significantly lower than that of both oviparous and hybrid females (Figure 2a). Clutch size was found to not differ significantly between parity modes (LRT: 2ΔLL = 4.1088, df = 2, *p* > .1). However, raw data indicates that oviparous females have on average 7 ± 2 eggs in a clutch and viviparous females have on average one fewer offspring (6 ± 2; Figure S2) than oviparous in a clutch. Hybrids were found to have around 7 ± 2 offspring per clutch.

Running speed and distance travelled were not affected by relative offspring mass (ROM) or parity mode. However, there was a significant interaction between ROM and parity mode for both average and maximum running speed (average speed *F*_2,78_ = 3.52, *p* < .05; maximum speed *F*_2,79_ = 2.99, *p* = .05). Average speed was found to decrease significantly with increasing ROM only for viviparous females (model estimate −51.60, *t* = −2.3, *p* = .024; Figure 2b; Table 1). This was also the case for maximum speed (model estimate −55.98, *t* = −2.14, *p* = .036; Figure 2c). There was no association between clutch size and the speed or distance travelled (*p* > .05 for all models). This shows that it is not the absolute number of individual offspring but the weight of surviving offspring relative to the mother's mass (ROM) that affects performance and does so only for viviparous females.

### 3.3 Effect of morphological traits and experimental procedure

There was a negative effect of female mass on the average speed, maximum speed, and average distance travelled, regardless of parity mode and pregnancy status (Table 1). Body length (snout‐vent length) affected speed and distance, with longer lizards sprinting faster (average speed *F*_1,108_ = 24.03, *p* < .001; maximum speed *F*_1,114_ = 18.28, *p* < .001) and travelling a greater distance (average distance *F*_1,118_ = 23.58, *p* < .001; maximum distance *F*_1,117_ = 7.94, *p* = .006; Table 1). There was no relationship between tail length and running speed or distance travelled. Both speed and distance were found to significantly decrease in the fourth trial (Table 1), which we attribute to habituation (similar to that observed in Husak et al., 2015).

## 4 DISCUSSION

Common lizards are active foragers that use fast sprint speeds and crypsis to escape predation (Bauwens & Thoen, 1981). Therefore, a reduction in running speed likely constitutes a survival cost: there is an increased risk of being caught by predators (Miles et al., 2000). Decreased running performance also results in an energy cost if it reduces the female's ability to capture prey (Qualls & Shine, 1998). Our finding that there is a locomotor cost of pregnancy in common lizards is consistent with results of other studies on squamate reptiles (Bauwens & Thoen, 1981; Bleu et al., 2012; Cooper et al., 1990; Dayananda et al., 2017; Goodman, 2006; Miles et al., 2000; Scales & Butler, 2007; Seigel et al., 1987; Shine, 1980, 2003b; Sinervo et al., 1991; Van Damme et al., 1989; Webb, 2004; Winne & Hopkins, 2006). However, there has been little investigation into whether the locomotor costs of pregnancy differ between oviparous and viviparous parity modes, especially while controlling for phylogeny and environmental variation. The evolution of reproductive strategies will be influenced by the associated costs and trade‐offs between life history and organismal performance traits (Husak & Lailvaux, 2022; Lailvaux & Husak, 2014, 2017; Reznick, 1985). Knowledge about these trade‐offs between parity modes provides new insight into the evolution and ecology of alternative parity modes.

We found that pregnancy had a negative effect on the running speed of female common lizards regardless of whether the lizard is oviparous, viviparous, or hybrid (Figure 1; Table 1). This primarily stems from the negative effect of the female's mass on the average and maximum speed as well as the state of being pregnant. These results support predictions that gravid females have a locomotor impairment due to the mass burden caused by pregnancy (Cooper et al., 1990; Le Galliard et al., 2003; Miles et al., 2000). On average, viviparous females were found to be heavier than oviparous females during pregnancy, in agreement with the theory that viviparous females incur a greater cost due to pregnancy (Recknagel & Elmer, 2019; Roitberg et al., 2013). However, viviparous females are not slower than oviparous females, despite their additional weight and the significant effect that weight has on running speed. This suggests that viviparous females compensate for the additional weight cost. This may be achieved by viviparous females being on average larger in body size than oviparous females, as lizard length has a positive effect on sprint speed (Table 1), or by having fewer and lighter offspring (as indicated by their lower ROM and clutch size; Figure 2; Figure S2). Although we did not find a significant difference in clutch size between oviparous and viviparous lizards in this study, this may be due to the smaller sample size of this dataset. Indeed, the values of clutch size and ROM used in this study are a subset of those used in Recknagel and Elmer (2019), which found that in this population of common lizards, viviparous females have on average one less offspring than oviparous females.

Loss of sprint speed performance due to pregnancy may be compensated for via a shift in behaviour, for example, by maintaining a crypsis strategy for longer before sprinting. This tactic was observed by Bauwens and Thoen (1981) for pregnant female viviparous common lizards compared to males and non‐gravid females. Our study found pregnancy had no effect on the average and maximum distance travelled by the females. We included this as a proxy measure of endurance, as there may be a reduction in distance travelled because of exhaustion due to the increased burden of the clutch as observed in other squamates (Cooper et al., 1990; Miles et al., 2000; Seigel et al., 1987; Zani et al., 2008). However, the lack of effect of pregnancy on these measures as we observed may be due to the fact that pregnant female common lizards are often located closer to their natural shelters and have shorter flight distances than non‐pregnant females (Bauwens & Thoen, 1981). This may suggest stronger selective pressure on pregnant females having a faster running speed as opposed to travelling further (for example, when crypsis fails).

We found that for viviparous lizards, reproductive investment had a significant effect on the average and the maximum speed (Table 1); viviparous females with lower ROM were faster (Figure 2b). This suggests a trade‐off between locomotor performance and reproductive investment, specifically for viviparous lizards, with increased reproductive investment negatively impacting locomotor performance. The additional locomotor cost associated with pregnancy for viviparous common lizards, irrespective of the physical burden due to increased mass, may result from an increased energy cost of pregnancy due to the late growth stages of the embryo (Bleu et al., 2012). This additional cost is incurred when the embryo starts to grow exponentially, increasing its metabolic oxygen demand and water uptake from the mother (Foucart et al., 2014). Oviparous females do not incur this cost because this stage of embryonic growth occurs external to the mother, in the egg after it has been laid (Qualls & Shine, 1998).

It has been suggested that in viviparous species in particular, additional physiological and endocrinological costs due to pregnancy may be present (Olsson et al., 2000), which we have not measured here. For example, a change in steroid hormones can alter short‐ and long‐term locomotor performance through effects on metabolic pathways or by muscle attrition (Le Galliard et al., 2003; Olsson et al., 2000). Additionally, it is thought that the balance of reproductive costs is dynamic over the duration of pregnancy, with oviparous females bearing greater burdens early in pregnancy and viviparous females bearing a greater burden at later stages of pregnancy (Bleu et al., 2012). Viviparous females have a lower reproductive output relative to oviparous females, and this may not just be constrained by physical cavity size but also by the additional cost of increased offspring mass on locomotion (Recknagel & Elmer, 2019). Our results suggest that viviparous females suffer from additional selective pressures during pregnancy relative to oviparous females: (i) the longer period of pregnancy and therefore longer period of reduced sprint speed, and (ii) additional offspring weight and physiological costs. While we predicted that these additional costs would negatively affect sprint speed, our experiments instead showed that viviparous females are not slower than oviparous females, indicating that they compensate for the costs (e.g., with larger body size).

Hybrid females were intermediate with respect to mass, reproductive investment, and sprint speed relative to oviparous and viviparous females (Figures 1 and 2a). This provides further evidence that reproductive costs vary between parity modes, with intermediate phenotypes resulting in intermediate costs. Given that the examined oviparous and viviparous females are syntopic and share similar environmental conditions, our finding suggests that reproductive traits are to a large degree genetically or epigenetically heritable. This agrees with previously conducted research showing that parity mode is a highly heritable trait in common lizards (Recknagel et al., 2021). Because hybrids generally displayed intermediate performance compared to each parental species in our experiment, hybrid inferiority that has been observed in other hybridizing lineages or species (Cullum, 1997; Denton et al., 2017; Mee et al., 2011—though see Kearney et al., 2005 for an example of increased performance in hybrids), does not seem to play a role in locomotor performance traits in common lizards. As the sample size for the hybrid individuals was lower than the oviparous and viviparous sample sizes, the statistical power for identifying significant differences was lower, and the conclusions we draw are therefore cautious. Further investigation into the life history and reproductive traits of hybrid individuals combined with genetic mapping would be beneficial to understand how and to what extent these traits are genetically determined. Additionally, testing the effect of female traits and reproductive investment on running speed in females, holding environment constant, could be tested across other viviparous lizard species to infer the universality of this relationship.

In conclusion, we found that: (i) there is a negative effect of pregnancy on locomotor performance in female common lizards (*Z. vivipara*) and that this is primarily due to their increased mass during pregnancy, (ii) there is no difference in locomotor performance between oviparous and viviparous common lizards in terms of average and maximum sprint speed or distance travelled, indicating that viviparous female lizards compensate to maintain performance; (iii) increased reproductive investment negatively affected locomotor performance in viviparous females only, suggesting a greater locomotor cost for viviparous females compared to oviparous, and (iv) hybrid individuals exhibited intermediate traits. Importantly, our study could robustly compare the reproductive and performance costs between these oviparous and viviparous lizards with minimal environmental and phylogenetic confounds. Future work into how reproductive costs vary among individuals and across the reproductive cycle will be valuable to elucidate proximate impacts on performance and ultimate consequences for the evolution of parity modes.

## AUTHOR CONTRIBUTIONS

**Robert Hussain:** Formal analysis (equal); investigation (equal); writing – original draft (lead); writing – review and editing (equal). **Hans Recknagel:** Conceptualization (equal); formal analysis (equal); funding acquisition (supporting); investigation (equal); writing – original draft (supporting); writing – review and editing (equal). **Kathryn R. Elmer:** Conceptualization (equal); funding acquisition (lead); investigation (supporting); supervision (lead); writing – review and editing (equal).

## FUNDING INFORMATION

K.R.E. received funding from Natural Environment Research Council NE/N003942/1 and K.R.E and H.R. received funding from Lord Kelvin‐Adam Smith PhD scholarship (Univ. Glasgow).

## CONFLICT OF INTEREST STATEMENT

We declare we have no competing interests.

## Supporting information


Figures S1‐S2.

## Data Availability

All the required data are uploaded as supplementary material [after acceptance, Data and scripts will be deposited in the University of Glasgow Enlighten Repository https://doi.org/10.5525/gla.researchdata.1158.
