# Supplementary material for: Higher locomotor costs of pregnancy in viviparous compared to oviparous common lizards (Zootoca vivipara)
Source: Ecol Evol. 2024 Aug 16;14(8):e70171. doi: 10.1002/ece3.70171 (PMC11327608; doi:10.1002/ece3.70171)

Supplementary Materials

**Figure S1.** Summary of Distance Locomotor Variables. Boxplots show the median value (thick line) and upper and lower quartiles, as well as the range of the locomotor variables **(A)** Average Distance and **(B)** Maximum Distance. Data taken from 106 female lizards (45 oviparous, 47 viviparous and 14 hybrids). Differences were not significant (average distance pregnancy status – LRT: 2ΔLL=0.326, df=1 p=0.568; average distance parity mode – LRT: 2ΔLL=1.368, df=2, p=0.505) except for maximum distance between oviparous post-parition and pregnant viviparous (model estimate -34.84, t =-2.47, p=0.014).

**
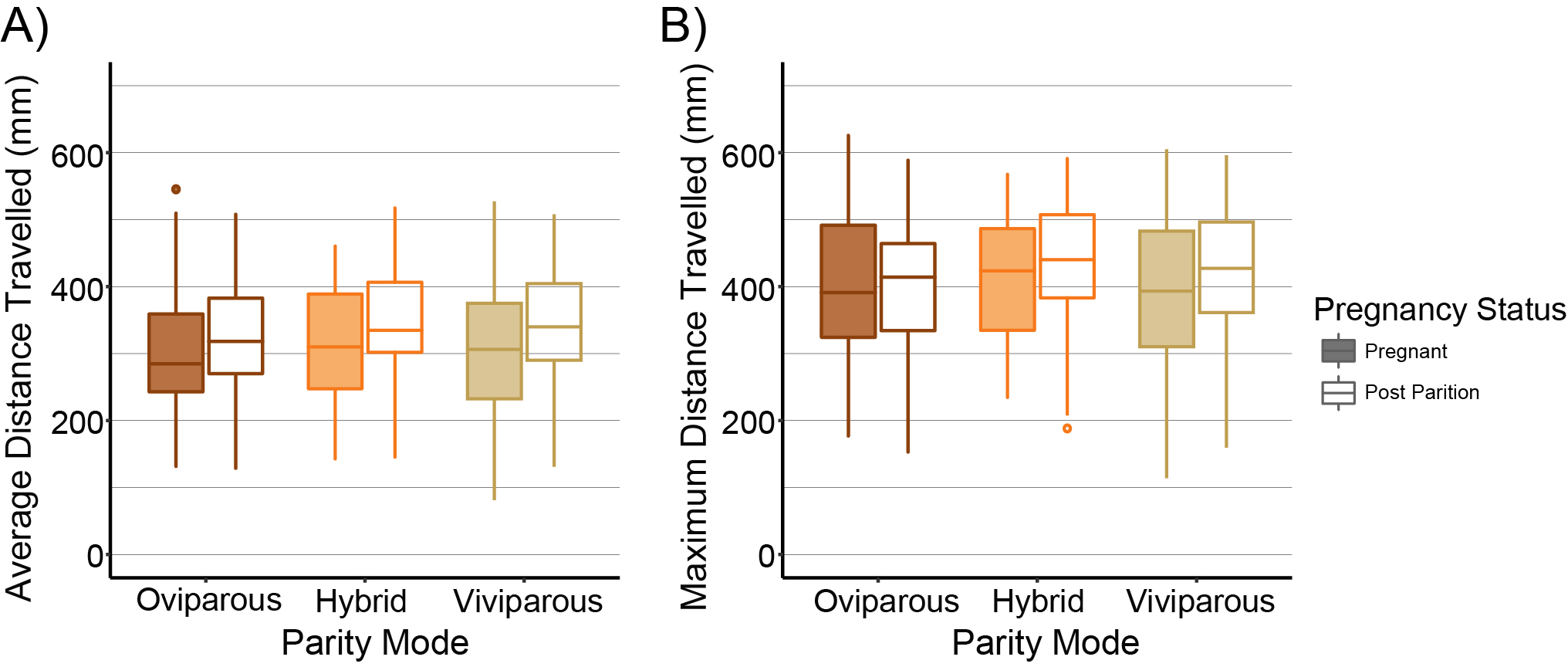
**

**Figure S2.** Boxplot of Clutch Size. median value (thick line) and upper and lower quartiles, as well as the range of the values of clutch size foreach parity mode. Data taken from 103 female lizards (42 oviparous, 47 viviparous and 14 hybrids). There is a significant difference in clutch size between oviparous and viviparous females (model estimate= -0.97, t=-2.34, p=0.021).


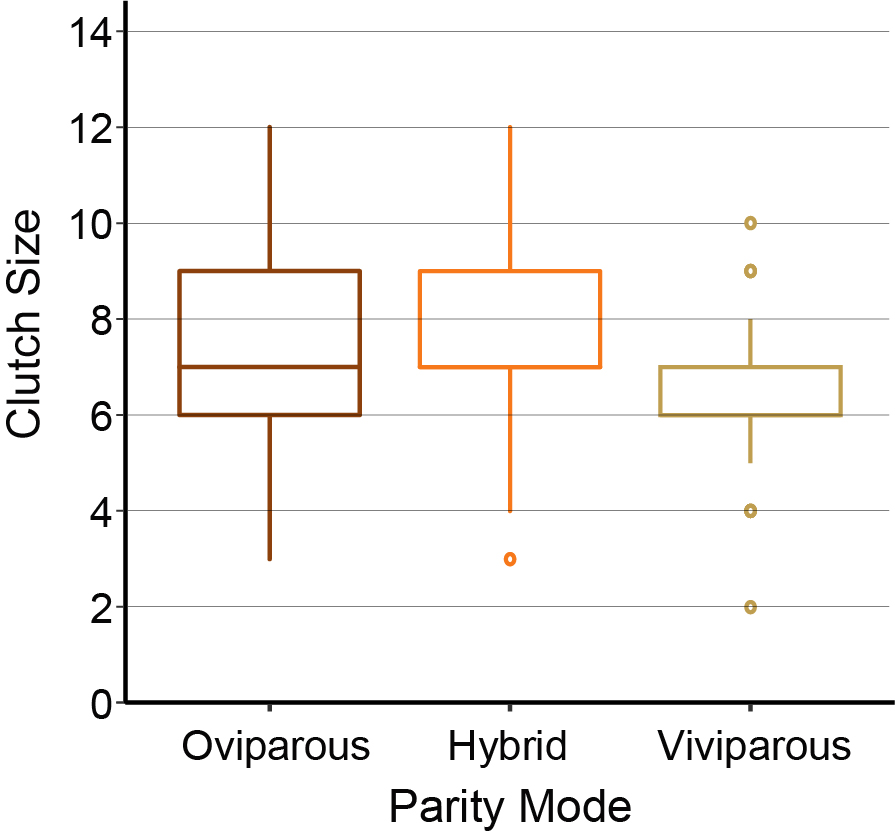

Supplement: Supplementary file 1 — Figures S1‐S2. [file ECE3-14-e70171-s001.docx]
